# Use of vector control to protect people from sleeping sickness in the focus of Bonon (Côte d’Ivoire)

**DOI:** 10.1371/journal.pntd.0009404

**Published:** 2021-06-28

**Authors:** Dramane Kaba, Vincent Djohan, Djakaridja Berté, Bi Tra Dieudonné TA, Richard Selby, Koffi Alain De Marie Kouadio, Bamoro Coulibaly, Gabehonron Traoré, Jean-Baptiste Rayaisse, Pierre Fauret, Vincent Jamonneau, Kouakou Lingue, Phillipe Solano, Steve J. Torr, Fabrice Courtin

**Affiliations:** 1 Institut Pierre Richet, Institut National de Santé Publique, Bouaké, Côte d’Ivoire; 2 Liverpool School of Tropical Medicine, Liverpool, United Kingdom; 3 Intertryp, IRD, Cirad, Univ Montpellier, Montpellier, France; 4 Programme National d’Elimination de la Trypanosomiase Humaine Africaine, Abidjan, Côte d’Ivoire; International Atomic Energy Agency, AUSTRIA

## Abstract

**Background:**

Gambian human African trypanosomiasis (gHAT) is a neglected tropical disease caused by *Trypanosoma brucei gambiense* transmitted by tsetse flies (*Glossina*). In Côte d’Ivoire, Bonon is the most important focus of gHAT, with 325 cases diagnosed from 2000 to 2015 and efforts against gHAT have relied largely on mass screening and treatment of human cases. We assessed whether the addition of tsetse control by deploying Tiny Targets offers benefit to sole reliance on the screen-and-treat strategy.

**Methodology and principal findings:**

In 2015, we performed a census of the human population of the Bonon focus, followed by an exhaustive entomological survey at 278 sites. After a public sensitization campaign, ~2000 Tiny Targets were deployed across an area of 130 km^2^ in February of 2016, deployment was repeated annually in the same month of 2017 and 2018. The intervention’s impact on tsetse was evaluated using a network of 30 traps which were operated for 48 hours at three-month intervals from March 2016 to December 2018. A second comprehensive entomological survey was performed in December 2018 with traps deployed at 274 of the sites used in 2015. Sub-samples of tsetse were dissected and examined microscopically for presence of trypanosomes. The census recorded 26,697 inhabitants residing in 331 settlements. Prior to the deployment of targets, the mean catch of tsetse from the 30 monitoring traps was 12.75 tsetse/trap (5.047–32.203, 95%CI), i.e. 6.4 tsetse/trap/day. Following the deployment of Tiny Targets, mean catches ranged between 0.06 (0.016–0.260, 95%CI) and 0.55 (0.166–1.794, 95%CI) tsetse/trap, i.e. 0.03–0.28 tsetse/trap/day. During the final extensive survey performed in December 2018, 52 tsetse were caught compared to 1,909 in 2015, with 11.6% (5/43) and 23.1% (101/437) infected with *Trypanosoma* respectively.

**Conclusions:**

The annual deployment of Tiny Targets in the gHAT focus of Bonon reduced the density of *Glossina palpalis palpalis* by >95%. Tiny Targets offer a powerful addition to current strategies towards eliminating gHAT from Côte d’Ivoire.

## Author summary

Gambian sleeping sickness (Gambian human African trypanosomiasis, gHAT) is a neglected tropical disease caused by *Trypanosoma brucei gambiense* transmitted by tsetse flies. Currently, Bonon is the focus which provides most cases of gHAT in Côte d’Ivoire. Screening and treatment of human cases has reduced the incidence of gHAT from 262 cases diagnosed between 2000 and 2004 to 24 cases during 2010–2015. We carried out a trial to assess whether Tiny Targets, insecticide-treated targets that attract and kill tsetse, could control *Glossina palpalis palpalis*, the most important vector of gHAT in Côte d’Ivoire. In 2015, we mapped human settlements, livestock, tracks, rivers and relict forest in Bonon and identified sites where humans may be bitten by tsetse. Monoconical (“Vavoua”) traps were deployed at these sites to provide an estimate of the abundance of tsetse. Between 2016 and 2018, ~2,000 Tiny Targets were deployed annually across Bonon and the impact of Tiny Targets was evaluated by changes in the numbers of tsetse caught by a network of 30 monitoring traps operated quarterly. In 2015, before deployment of Tiny Targets, the mean daily catch from the 30 monitoring traps was 6.4 tsetse/trap/day. Following deployment of targets, catches declined to <0.3 tsetse/trap/day representing a >95% reduction in tsetse abundance. Between February 2016 and December 2018, no recent (Stage 1) cases of gHAT have been reported in Bonon. Our results demonstrate that Tiny Targets can contribute to the elimination of gHAT through tsetse control. Tiny Targets have been adopted as an important tool in Côte d’Ivoire’s national strategy to eliminate gHAT.

## Introduction

Gambian Human African Trypanosomiasis (gHAT), or Gambian sleeping sickness, is a vector-borne disease found in West and Central Africa, caused by *Trypanosoma brucei gambiense* transmitted to humans by the bite of an infected tsetse fly (*Glossina* sp.) [1]. There are no vaccines or preventative drugs for this lethal disease and treatment is complex, involving at least two weeks hospitalisation during chemotherapy, albeit recent advances have produced a novel orally administered treatment (Fexinidazole, 1-Methyl-2-((4-(methylthio)phenoxy)methyl)-5-nitro-1H-imidazole, chemical formula: C12H13N3O3S) which has reduced hospitalisation for treatment to under 10 days [2].

The World Health Organization (WHO) aimed to eliminate gHAT as a public health problem by 2020. Specific targets included reducing annual incidence to less than one new case per 10,000 people in at least 90% of endemic foci, and a global total of less than 2,000 new cases reported per annum [3]. Over the past decade, the annual number of cases reported by WHO shows a consistent global decline [4]. Most recently published figures show there were less than 1,000 cases of gHAT reported in 2018 (953 cases) and 2019 (864) compared to >10,000 cases/year prior to 2009 [2, 5].

In Côte d’Ivoire, the annual number of gHAT cases has fallen from 326 in 1995 to eight cases in 2010 [3]. Surveillance of cases was affected by civil warfare between 2002 and 2010, but with the re-establishment of sustained peace in 2011, medical screening activities resumed [6]. Since resuming, 35 gHAT cases were detected between 2011 and 2015, most of these originated from either Bonon or Sinfra foci [7]. This low incidence of gHAT suggests that the WHO elimination goal is within reach for Côte d’Ivoire.

Historically, control operations against *Glossina palpalis palpalis* have contributed to the containment of gHAT outbreaks, as in the Vavoua focus. These interventions relied on the deployment of insecticide-impregnated monoconical traps [8, 9]. The Vavoua focus was identified in 1975 by the medical surveillance team from Centre Muraz in neighbouring Burkina Faso, when the prevalence of gHAT was 1.7% [10] but the focus remained active until 1982 [11]. In November 1983, monitoring traps caught 4 tsetse/trap/day ahead of the deployment of insecticide-impregnated Vavoua traps, following which the catches reduced to 0.64 tsetse/trap/day in July 1984 [12]. A secondary tsetse control campaign was conducted between November 1987 and November 1989, reducing tsetse catches to 0.01 tsetse/trap/day [13]. gHAT surveillance of the human population prior to the beginning of the second tsetse control campaign revealed a prevalence of 0.05% (11 positive from 21,705 people screened), and after 24 months of additional tsetse control, another medical survey of 6,742 reported no cases [8, 13]. Superficially, the absence of cases in the second survey might suggest that gHAT has been eliminated but consideration of the 95% confidence limits [14] suggests that the true rates is ≤0.044%, which is only slightly less than the initial figure.

In the last decade, a more cost-effective tsetse control method has been developed using Tiny Targets reducing the annual cost of intervention to USD 66-86/ km^2^ [15, 16]. In West Africa, these comprise 0.375 m^2^ (75 x 50 cm) panels of insecticide-impregnated blue polyester fabric flanked by panels of black polypropylene net [17]. Attracted tsetse contact the target, picking up a lethal dose of insecticide [18]. Implementation of control using Tiny Targets have been conducted successfully in Chad, Uganda, Democratic Republic of Congo and Guinea where *Glossina fuscipes fuscipes*, *Glossina fuscipes quaziensis* and *Glossina palpalis gambiensis* are the principal vectors [19–21].

Since the end of the 1990s, the Bonon gHAT focus is the most active in Côte d’Ivoire [22–24]. More recently, from 2000 to 2015, 325 gHAT cases were diagnosed, showing that transmission is still occurring and that Bonon remained the principal focus of gHAT in Côte d’Ivoire [7, 25]. Here we assess the first utilisation of Tiny Targets in Bonon against *G*. *p*. *palpalis*, one of the most important vectors of gHAT in West Africa [26].

## Methods

### Ethical statement

Ethical clearance for this work was granted by the Comite National D’Ethique De La Recherche (CNER) of the Ministere De La Sante Et De L’Hygiene Publique—Côte d’Ivoire. Approval reference number: 030-18/MSHP/CNER-kp. Formal consent for study inclusion was not obtained prior to the collection of census survey data from each respondent, this was to ensure the anonymity of participants.

### Study area

**Fig 1:**
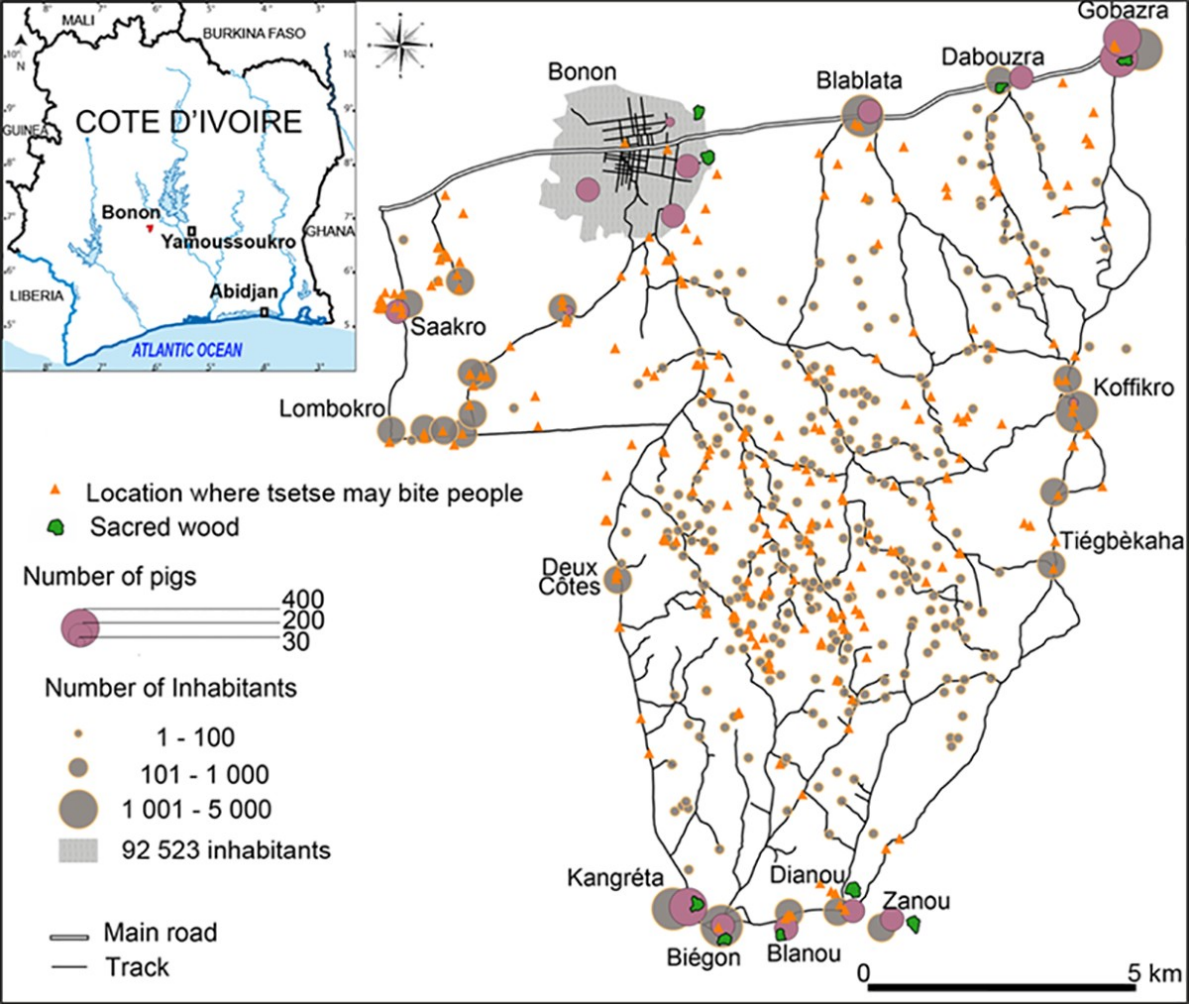
Location of the study area, settlement and sites where tsetse-human contact is relatively high.

The study area is in Marahoue region, located 100 km west of the capital, Yamoussoukro. The tsetse control intervention area was ~130 km^2^ in size covering both urban (Bonon town) and rural (villages, hamlets, encampments) populations (Fig 1). The dominant ethnic group in the intervention area are the Gouro people and other groups (Baoule, Mossi, Senufo, Lobi, Malinke, Bobo) have immigrated to the area from elsewhere in Côte d’Ivoire and neighbouring countries (principally Burkina Faso and Mali) [24]. Environmental characteristics of the area was historically dominated by mesophile forest which has been largely deforested and replaced by cash crop plantations (coffee and cocoa) alongside subsistence crops such as banana and rice between 1970 and 2015 [24, 27]. The deforestation observed in Bonon seems to have led to the disappearance of tsetse species (*Glossina fusca*, *Glossina pallicera*, *Glossina nigrofusca*) usually present in well conserved forest area [28], and only one tsetse species (*Glossina palpalis palpalis*) was able to survive to environmental change provoked by deforestation [27]. Mesophile forest was already destroyed before commencing this tsetse control intervention, excepting sacred woods that are located at the periphery of villages [27]. Livestock production is commonplace with pigs and cattle being the principal livestock species [29].

**Fig 2:**
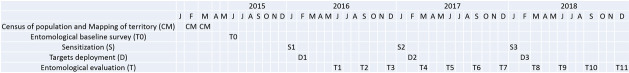
Timeline of activities undertaken in the intervention area between February 2015 and December 2018.

Bonon focus was formed from the historical focus of Bouafle which extended 60 kilometres to the east of Bonon. The introduction of gHAT into Bonon was the result of people from Bouafle seeking work in Bonon [30, 31]. gHAT became endemic in Bonon during the 1950s, with nine cases detected between 1956 and 1959, raising to 57 between 1976 and 1985, then 125 diagnosed between 1986 and 1997 [22]. Medical active screening surveys conducted during 1998, 1999 and 2000 detected 129 cases from 22,824 people screened (0.64%) [27]. All these cases were identified from Bonon town and the rural area located immediately to its south; none of these cases originated from north of Bonon town [23, 24]. Responding to the clear pattern of gHAT case origin, we aimed to deploy Tiny Targets within the town of Bonon and the rural area to its south. The sequence of vector control activities carried out between February 2015 and December 2018 is summarised in Fig 2.

### Census of population and mapping of the intervention area

In February and March 2015, the geo-location of each settlement was recorded using Global Positioning System (GPS) and a structured form was completed during an interview with each settlement’s chief, obtaining data on the number of people residing in the rural area, these data are shown in Fig 1. Settlements were classed as encampments (1–100 inhabitants), hamlets (101–1000 inhabitants) or villages (1001–5000 inhabitants), according to population size. Data on livestock animal population (pigs, cattle) in each settlement were also obtained. All roads and paths in the intervention area were recorded by GPS. Locations where tsetse may feed on humans (e.g., watering points, track/river intersections, livestock corrals, swimming/fishing points and remnants of natural forest habitat) were also geo-referenced.

### Baseline entomological survey T0

In June 2015, a pre-intervention entomological survey was carried out with monoconical “Vavoua” traps deployed at 278 sites that were identified during the census as being places where probability of tsetse biting humans is relatively high (Fig 2). Traps were operated for 48 hours and tsetse collected and counted at the end of this period. From these 278 trap locations, a subset of 30 sites were selected to be monitoring traps to assess the impact of Tiny Targets on tsetse abundance, infection and distribution. The 30 sites were selected considering tsetse densities, infection rate and the spatial coverage of the control area. The absence of non-control trap site in the surrounding of the Bonon tsetse control intervention area, render it impossible to evaluate changes to tsetse population that did not receive vector control, and so to evaluate the potential impact of other parameters such as climate and vegetation changes, on the evolution of tsetse density in our tsetse control intervention area.

### Sensitization

In January 2016, 2017 and 2018, prior to each deployment of Tiny Targets, the population of the intervention area was provided with information on the purpose of Tiny Targets (Fig 2). State and town authorities, chiefs of settlements and community leaders were visited first, followed by schools. Spot radio announcements, describing the activity of the team in French, Gouro, Malinké, Mossi, Baoulé and Lobiri languages, were broadcast on the local radio station (radio “Concorde”) for ten days before target deployment. These announcements were also broadcast with a megaphone in markets, along main tracks and major settlements. Team members wore T-shirts, featuring images of tsetse flies and Tiny Targets.

### Tiny Target mediated tsetse control

Tiny Targets measuring 75cm wide by 50cm high (0.375 m^2^ total area) were deployed to reduce the tsetse population. Target construction features a 45cm wide by 50cm high central panel of light blue (“Vestergaard blue”) polyester, flanked by 15cm wide by 50cm high panels of black polypropylene netting impregnated with deltamethrin (300 mg/m^2^) [32]. Targets were obtained commercially from Vestergaard-Frandsen (Lausanne, Switzerland). In February 2016, the first deployment of Tiny Targets was implemented, guided by information gathered during the above-mentioned census and mapping survey (February to March 2015) in combination with tsetse catch data from the T0 entomological baseline completed in June 2015 (Fig 2). The second and third deployments of Tiny Targets were carried out in February 2017 and February 2018 respectively.

### Entomological monitoring

Following the T0 and deployment of Tiny Targets, eleven monitoring surveys (T1-T11) were carried out at three-month intervals quantifying changes in the tsetse population (density and infection). Fig 2 details this timeline. Each monitoring session used the same 30 trapping sites, Vavoua traps were operated for 48 hours. For the final survey (T11) conducted in December 2018, we deployed traps at 274 sites—including the 30 sites used for all T1-T10 surveys, matching the locations used for the initial T0 entomological baseline survey. This reconstruction of the full T0 survey provides a comprehensive overview before and after vector control.

### Calculation of trypanosome infection rate in tsetse population

Collected tsetse were identified morphologically and then dissected using a stereo microscope (Zeiss, Stemi DV4) to isolate the proboscis, midgut and salivary gland which were placed on slides [8]. Isolated organs were examined using an optical microscope (Novex B, led Series) at 100 and 400 magnification for trypanosomes presence.

No molecular tests were used to identify the trypanosome species, so it is not possible to specify the trypanosomes species involved in tsetse infection.

### Statistics

Tsetse catches were analysed using generalized linear mixed-effects models (GLMM) with a negative binomial error distribution. Trap locations were specified as random effects and the sampling round (T0-T11) and presence or absence of Tiny Targets were fixed effects. All analyses were carried out using the R package glmmADMB [34]. Traps were operated for 48 hours. To aid comparison with wider literature, we also report the mean daily catch of tsetse/trap, calculated by halving the mean catch/trap estimated from the GLMM. Proportions of tsetse infected with *Trypanosoma* were also analysed using GLMM with a binomial error distribution in which the number of tsetse dissected was the binomial denominator. The statistical significance of differences in catch or proportion infected was assessed using Tukey’s multiple range test using the multcomp package.

## Results

### Census of population and mapping of the intervention area

In addition to Bonon town itself (92,523 inhabitants according to the national census in 2014), 331 settlements were recorded comprising 11 villages (22,230 inhabitants), 12 hamlets (3,035 inhabitants) and 308 encampments (1,432 inhabitants) (Fig 1). A total of 326 locations that were potential sites for tsetse biting were identified, including 212 watering points, 91 intersections between tracks and river, 11 livestock enclosures (pigs and cattle) and 12 locations where people participate in fishing and swimming. A total of 2,471 pigs (predominantly free ranging) and 1,710 cattle were recorded, mainly in Bonon town and larger villages.

### T0 entomological baseline survey

**Fig 3:**
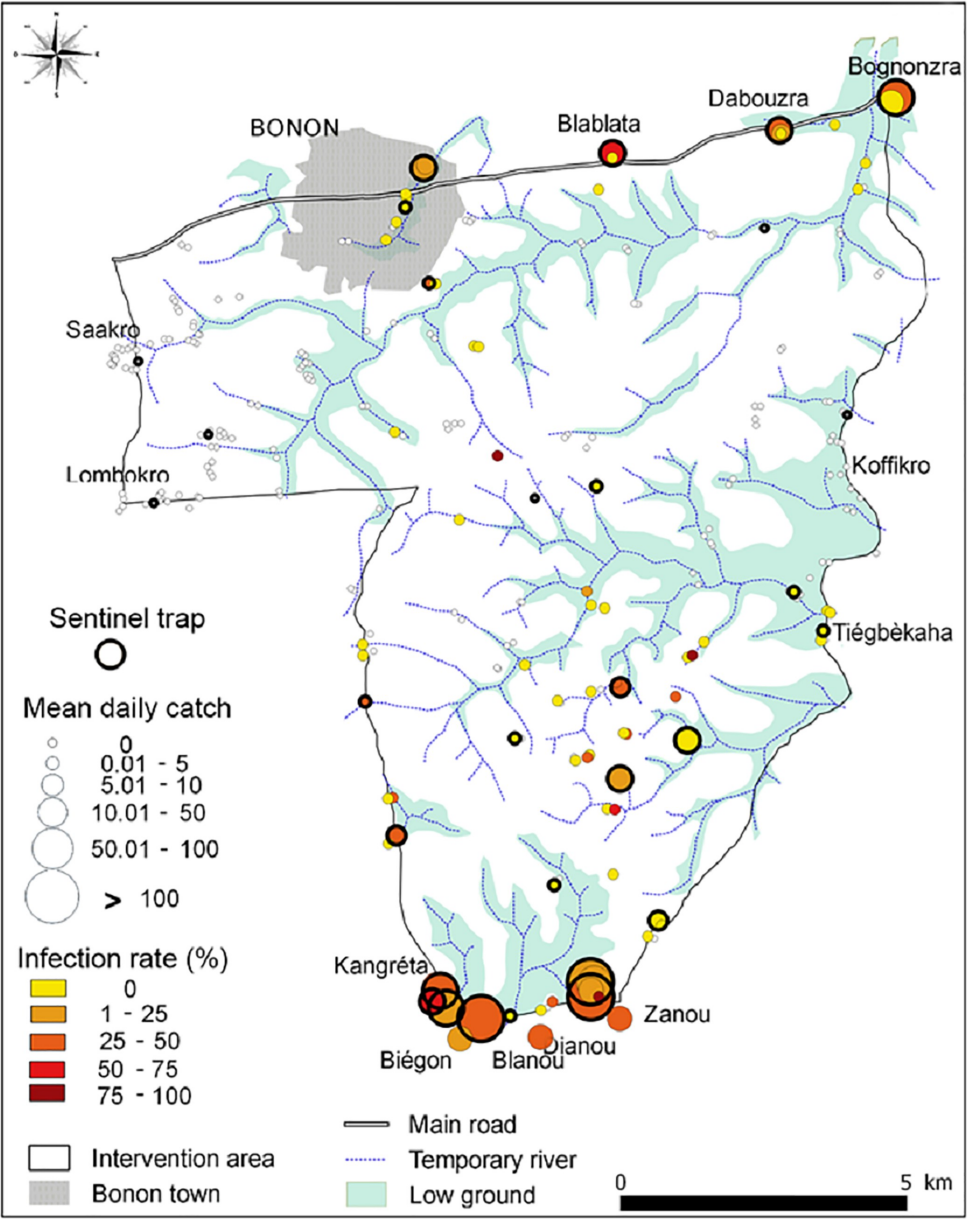
Tsetse densities and infections of the T0 baseline entomological survey.

During the T0 baseline survey, a total of 1,909 *G*. *p*. *palpalis* were caught from 278 unique trap locations (median catch = 0 tsetse/trap, range = 0–259) operated for two days giving a mean catch of 0.013 (0.003–0.050, 95% CI; 0.007 tsetse/trap/day). Of the flies caught, 437 were analysed by dissection and 101 (23%) were infected with *Trypanosoma*. Tsetse were more numerous along the river which traverses Bonon town, around main Gouro autochthonous villages (Blablata, Dabouzra, Bognonzra, Kangreta, Biegon, Blanou, Dianou, Zanou) and in the central rural area (Fig 3). Of the 278 traps, 81 caught at least one tsetse and 35 traps caught at least one tsetse infected by *Trypanosoma*. The mean catch of the 30 sentinel traps was 12.7 tsetse/trap (5.04–32.20, 95%CI), i.e., 6.3 tsetse/trap/day, the median catch was 10 (range 0–259) and of the 245 tsetse from this sample examined for trypanosomes, 66 (8.4%) were infected.

### Sensitization

Large scale sensitization campaigns were carried out before targets were deployed. Overall, the message was given to 99 community leaders, of whom 26 were school staff, 17 religious leaders, 42 chiefs of settlement and 14 co-operative associations (cattle breeder, agricultural). The field team also disseminated information directly to 6,833 individuals that were encountered opportunistically during operations in the intervention area. The number of people informed through the local station “Radio Concorde” is undefined.

### Targets deployment

**Fig 4:**
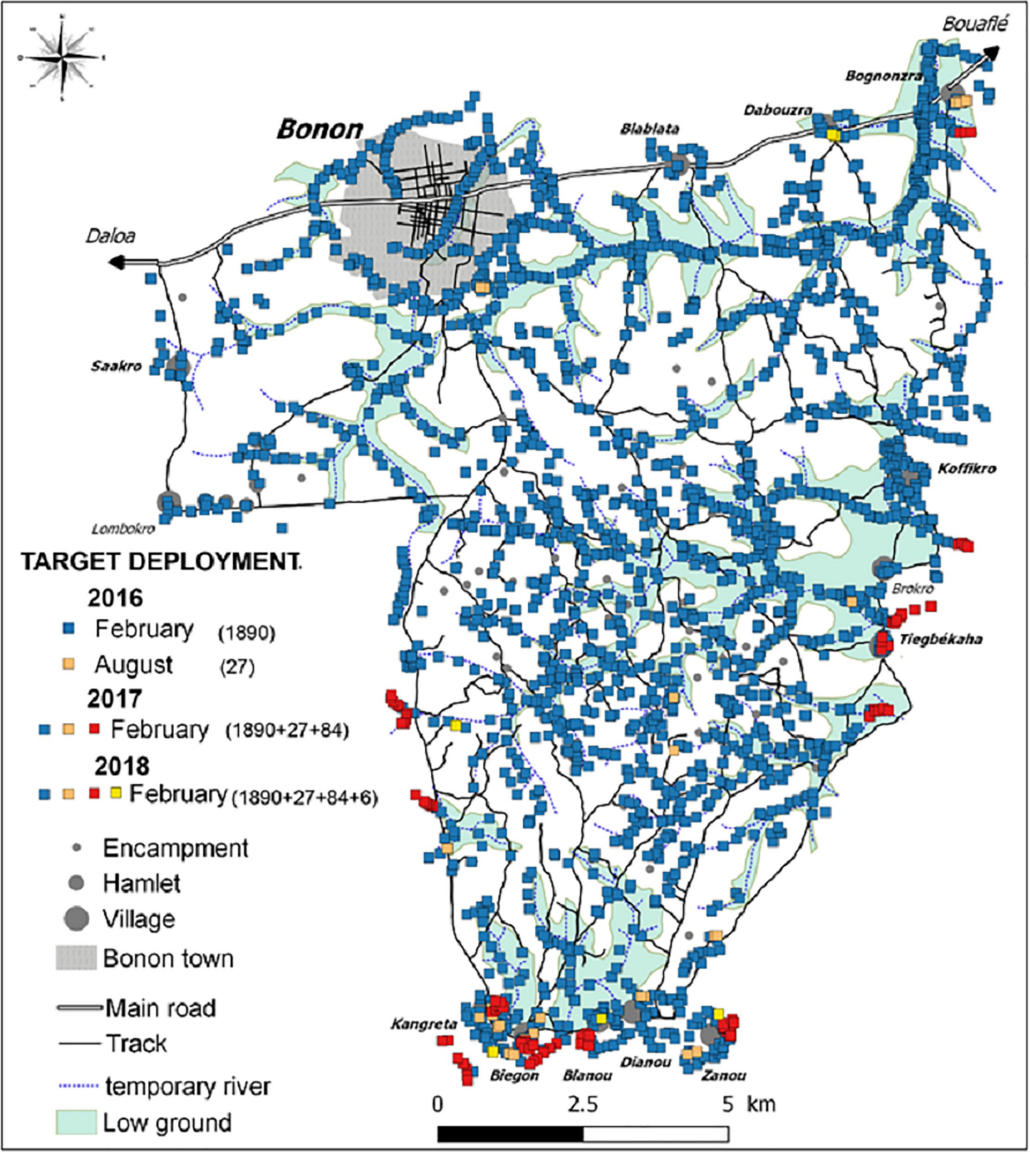
Location of Tiny Targets deployed in the intervention area in 2016, 2017, 2018.

In February 2016, 1,890 Tiny Targets were deployed throughout the intervention area. In August 2016, an additional 27 targets were added to reinforce control in places where the T1 round of monitoring had revealed that the tsetse population persisted. In February 2017 (second deployment), all the previous targets (1,890 + 27) were replaced along with a further 84 additional targets, responding to areas where monitoring data showed tsetse were present. In February 2018 (third deployment), all targets were replaced (1,886 + 27 + 84) with the addition of six new target sites, giving a total of 2,003 Tiny Targets deployed in the intervention area. This equates to ~15 Tiny Targets / km^2^ across the intervention area, or one Tiny Target for every 59 inhabitants of the intervention area. The positioning of Tiny Targets is shown in Fig 4.

### Entomological monitoring

**Fig 5:**
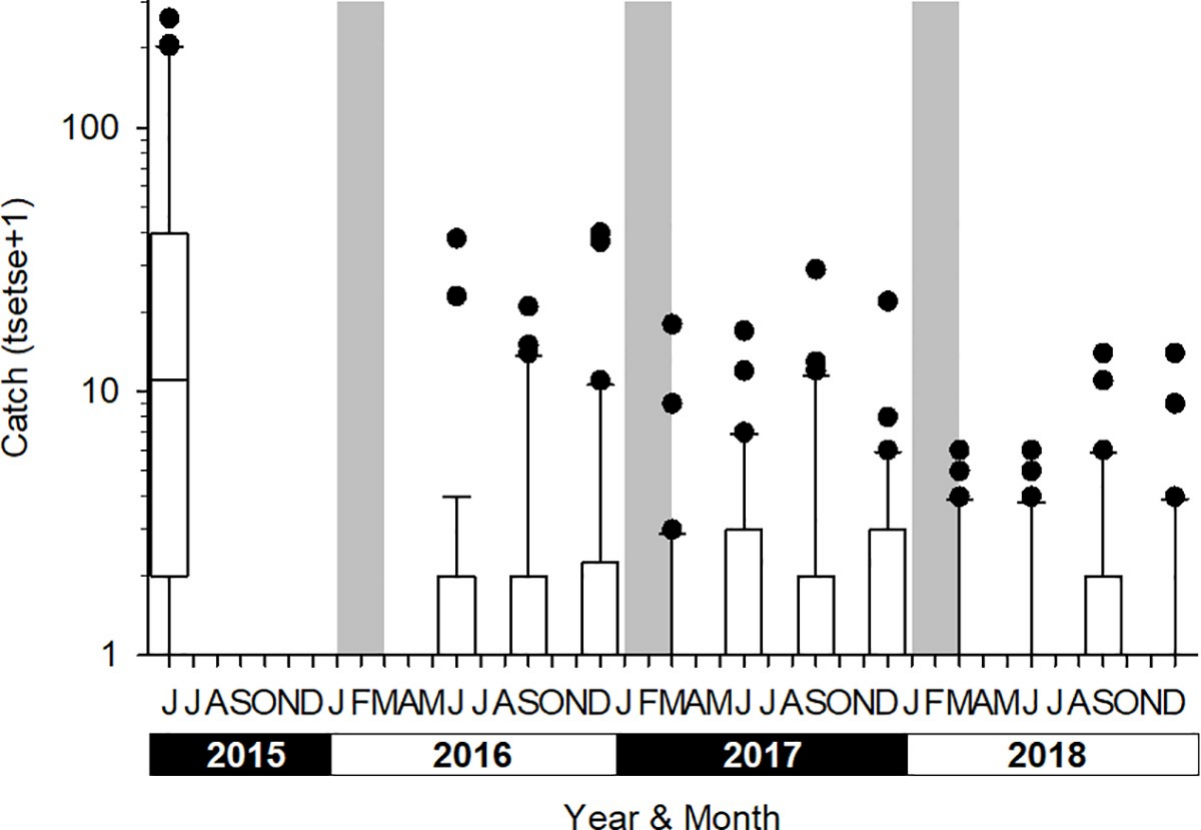
Boxplot of the catch of tsetse from 30 monitoring traps, each operated for 48 h, before and after Tiny Targets were deployed (grey vertical bars) in Bonon. The boxplot shows the median and interquartile range, whiskers show the 10th and 90th centiles and the black circles show all values >90th centile.

**Fig 6:**
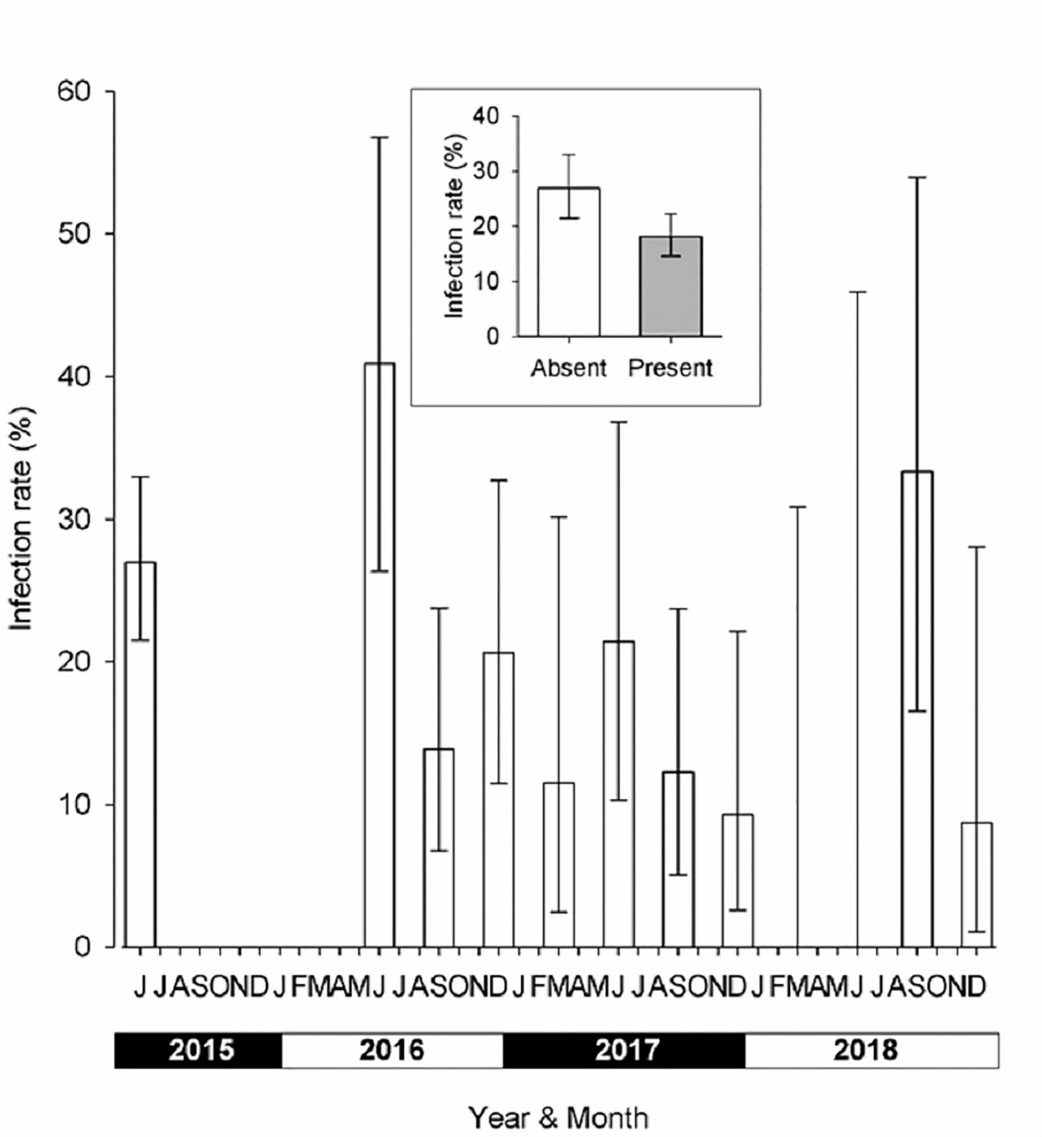
Percentage (±95%CI) of tsetse infected with trypanosomes. Inset shows percentages when targets were absent or present from the intervention area.

**Fig 7:**
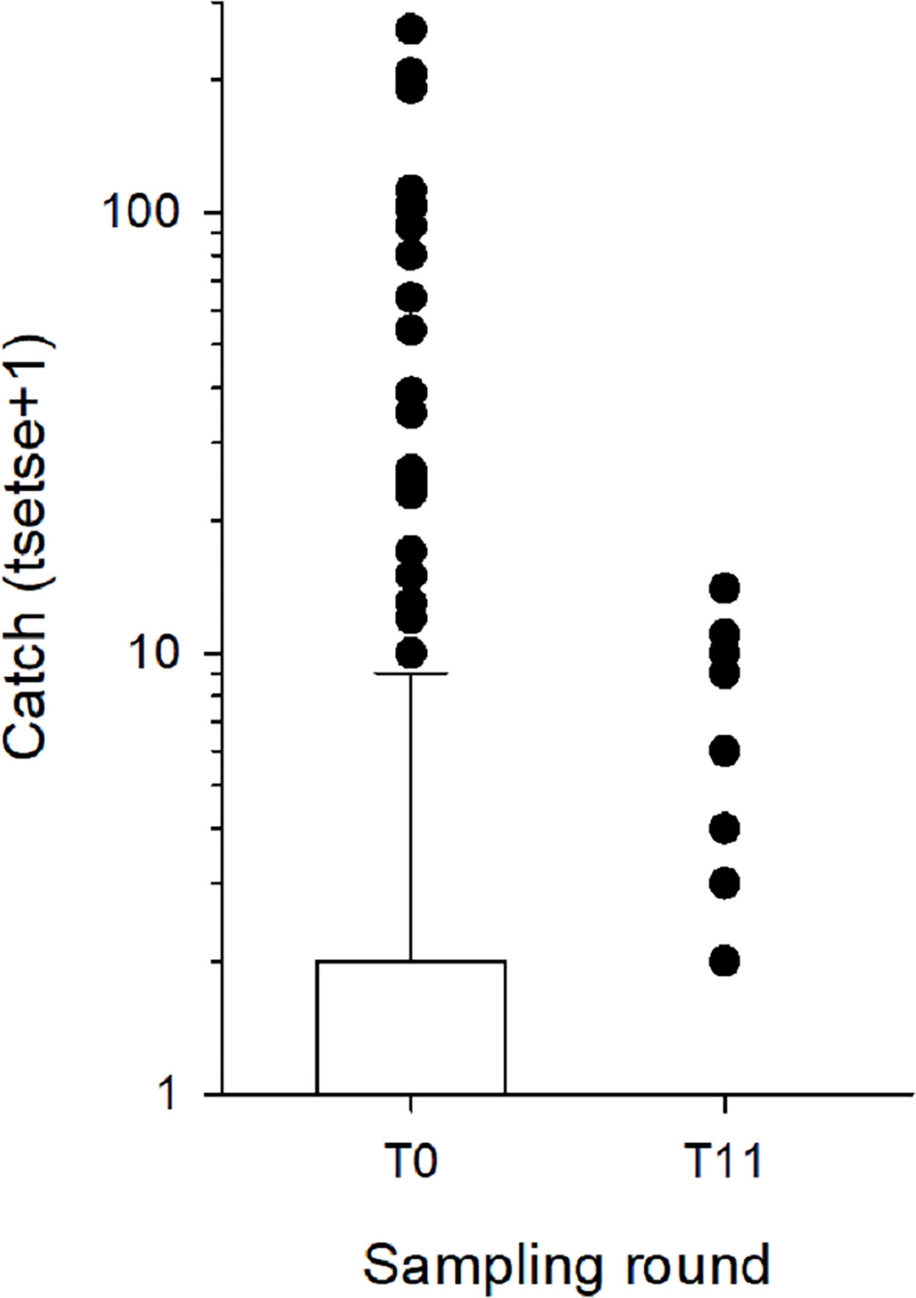
Boxplot of the catch of tsetse from 274 monitoring traps deployed for 48 h at identical sites in June 2015 (T0), before targets were deployed, and December 2018 (T11) after three rounds of target deployment. The boxplot shows the median and interquartile range, whiskers show the 10th and 90th centiles and the black circles show all values >90th centile.

**Fig 8:**
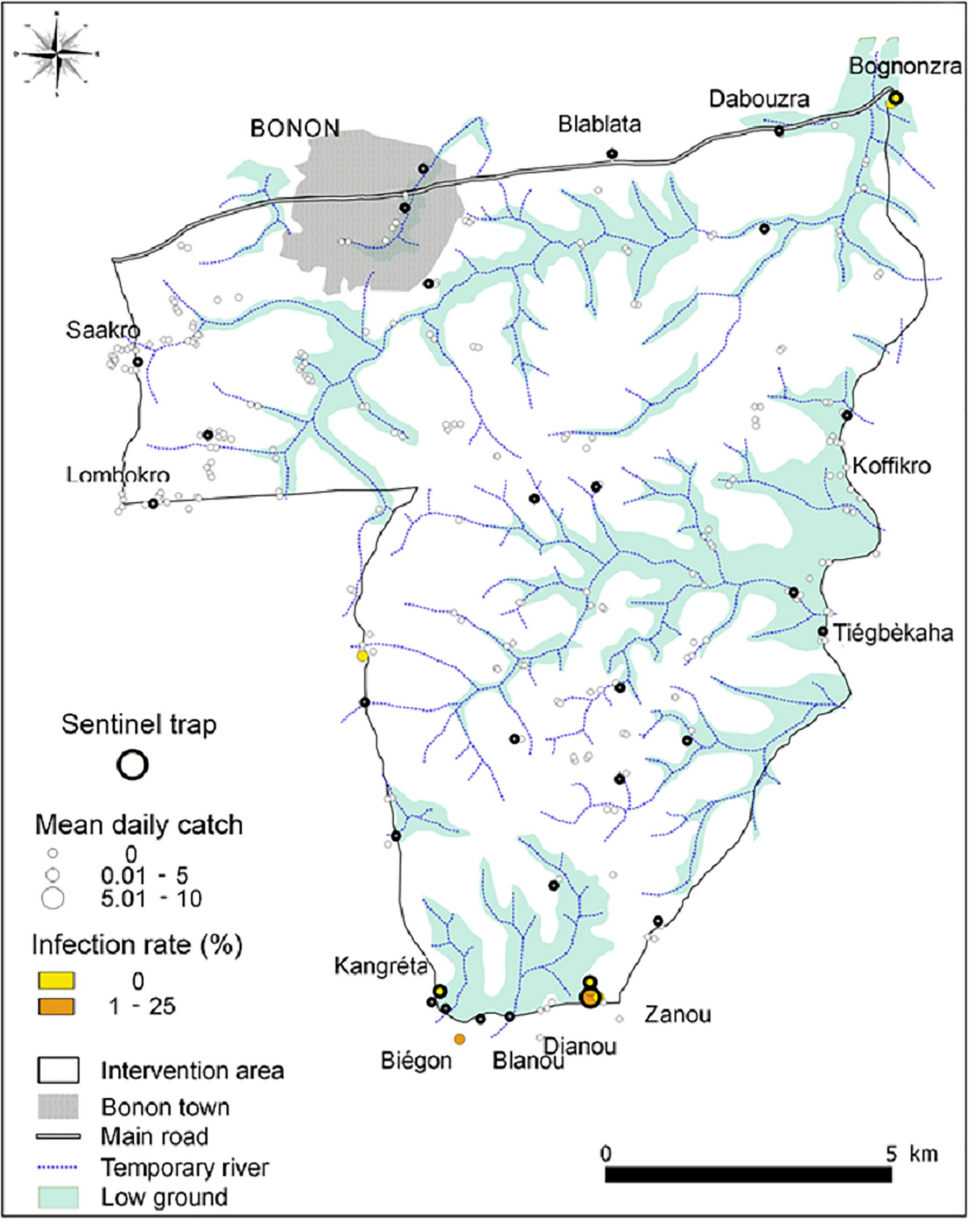
Numbers and infection rates of tsetse caught during the T11 entomological survey conducted in December 2018.

Boxplots of the catches from each sampling round are shown in Fig 5. The first post intervention monitoring evaluation (T1) followed the deployment of Tiny Targets by three months, and the mean catch from the 30 sentinel traps was 0.4 tsetse/trap (0.13–0.47, 95%CI; 0.2 tsetse/trap/day) compared to 12.7 tsetse/trap (5.05–32.20, 95%CI; 6.4 tsetse/trap/day) at T0. Mean catches remained low in all subsequent surveys with catches ranging between 0.1 (0.02–0.26) and 0.6 (0.17–1.79) tsetse/trap, *i*.*e*. 0.05–0.3 tsetse/trap/day. There was no significant difference (*P* = 0.052–1.000 for all possible contrasts) in catches between T1 and T11 but all were significantly (*P*<0.001) less than that at T0. Overall, the mean catch following the deployment of targets was 0.24 tsetse (0.07–0.80, 95%CI; 0.1 tsetse/trap/day) representing a 98% (0.24/12.7) decline in comparison to the catches at T0. In terms of infection, Fig 6 demonstrates the observed decrease in infection rates in tsetse caught by the 30 sentinel traps from 28.4% (66/245) at T0 to 8.7% (2/23) at T11, the figure’s inset contrasts the infection rates before deployment of Tiny Targets with the findings following the initial deployment of February 2016. There was no significant difference (P>0.05) in infection rates between sampling rounds, but the low numbers of tsetse caught following the deployment of targets reduced sample sizes and hence increased confidence intervals. To overcome this limitation, we compared infection rates of tsetse caught before targets were deployed (T0, 245 tsetse) and after (T1-T11, 413 tsetse) using a GLMM with site and sampling round (T0-T11) specified as random effects. The infection rates before and after deployment of targets were 26.2% (11.29–49.69) and 15.2% (3.21–49.36), respectively, but the difference was not significant (P>0.05). Without molecular analysis we are unable to distinguish *T*. *brucei* from *T*. *b*. *gambiense* infection or to another trypanosome species, so the detected reduction of infection in trypanosomes in the tsetse population applies to all trypanosome species. During the T11 entomological survey 274 traps were set up in the same locations used during the initial T0 survey, 52 tsetse were caught compared to 1,909 at T0 (Fig 7, P<0.001 for difference between means). A total of 43 (43/52 = 82.6%) tsetse were examined for trypanosomes and five (5/43 = 11.6%) were infected, compared to T0 (1909/437 = 4.4% examined, 101/437 = 23% infected). While the percentage of infected tsetse at T11 (11.6%) was lower than that at T0, the difference was not significantly different (P>0.05). Fig 8 shows the catches of tsetse and trypanosome infection rate, demonstrating that the distribution of the few remaining tsetse are spatially limited when compared to before control. Tsetse are now associated with villages located at the southern or northern edges of the intervention area.

## Discussion

### Impact of control on tsetse abundance and trypanosome transmission

Following three years of tsetse control using Tiny Targets (February 2016 to December 2018), the mean daily catch of *G*. *p*. *palpalis* from a network of 30 sentinel traps declined by >95%. Detailed comparison of a more extensive network of traps operated at T0 (278 traps, 1,909 tsetse caught, 101/437 infected) and T11 (274 traps, 52 tsetse caught, 5/43 infected) further supports the finding that Tiny Targets reduced the abundance of tsetse across Bonon gHAT focus by >95%. Infection rates were generally lower following the deployment of targets, but not significantly so. The impact of Tiny Targets on *G*. *p*. *palpalis* in Bonon is similar to that seen in gHAT foci in Uganda, Guinea and Chad [19–21].

Following deployment of Tiny Targets there was a marked change in the spatial distribution of tsetse. The changes in catches of tsetse from traps suggest that tsetse declined and sometimes disappeared in Bonon’s urban areas where historically most cases of gHAT originated [23]. These results suggest that Tiny Targets will have greatly reduced the entomological inoculation rate of tsetse to humans and, in turn, trypanosome transmission in these urban areas. gHAT cases had also previously occurred in rural areas south of Bonon [24]. Here too there was a marked decline in tsetse catch suggesting that the biting risk from tsetse was reduced. We stress that while no tsetse were caught in some areas following the deployment of Tiny Targets, the absence of tsetse in traps does not mean that tsetse have been eradicated locally.

During the tsetse control operation, three cases of gHAT occurred in the focus of Bonon. All these cases were from the intervention area (one person in Bonon town, another to Tiegbékaha hamlet and one to Biegon village). These cases were all in second stage of gHAT (all have received effective medical treatment) and it is most likely that they were infected before targets were deployed. Nevertheless, the detection of cases in Bonon after Tiny Targets were deployed suggests that gHAT is still present and vector control can contribute to slow down trypanosome transmission, including *T*. *b*. *gambiense*. During the time that case detection and treatment remains active, there is a strong supposition that the addition of vector control capitalises on declining gHAT cases. While we have no unequivocal evidence that vector control has successfully interrupted transmission, published modelling work [33, 34] exemplify that vector population control between 60 or 90% is sufficient to interrupt transmission to humans, putting this control operation’s impact of over 95% well above the modelled thresholds for interruption. In the context of gHAT elimination, appropriately implemented tsetse control contributes by ceasing transmission while the infected persons within a focus are identified and treated by medical systems. This strategic limitation of transmission not only adds protection for focus residents but also reduces overall timeframe and costs of gHAT elimination with cost benefits to doners.

The heterogeneous distribution of livestock, principally pigs and cattle, in the focus of Bonon is relevant as tsetse feed regularly on these species in forested areas of Côte d’Ivoire [35, 36]. Livestock in these areas are not only at risk of animal African trypanosomosis (AAT) but may also act as hosts for *T*. *b*. *gambiense* [29]. It may therefore be beneficial to treat pigs and cattle with insecticide adopting a One Health approach, as was done with pigs on the Loos islands of Guinea [37] or cattle in the *T*. *b*. *rhodesiense* focus of Tororo in Uganda [38]. Authors acknowledge that insecticides can be topically applied to pigs, with impact upon tsetse but free ranging pigs are difficult to restrain for treatment [39, 40]. Further complications come from the time taken for porcine livestock to mature to slaughter being rapid, especially when compared to cattle, this rapid turnover often translates to owners being less willing to invest in treatments for the shorter-lived livestock [41]. Locations where trypanosome infected tsetse were caught during the T11 survey are all in or close to pig keeping villages, suggesting that trypanosome infected domestic pigs are driving the infection of these tsetse. As the husbandry of Bonon’s pig population is a mix between free range and penned systems, it could be possible to strengthen tsetse control in specific problematic areas by using Insecticide Treated Netting (ITN), around present pigsties. The impact of ITN treated sties on tsetse populations has been successfully demonstrated in Ghana [42].

### Sustainable tsetse control to achieve the interruption of *T*. *b*. *gambiense* transmission

Our work in Bonon is not the first implementation of tsetse control in a gHAT focus of Côte d’Ivoire and lessons can be learned from previous experiences. Historic records of gHAT cases reported by the passive screening centre located in Daloa focus (Programme de Recherche Clinique contre les Trypanosomiases, PRCT) can be compared for Vavoua and Sinfra foci since the end of the vector control campaigns, providing applicable information, as the two areas have had differing impacts.

In Vavoua, the tsetse control implemented in the 1980s produced a marked decline in tsetse densities, from 4 tsetse/trap in November 1983 to 0.01 tsetse/trap in November 1989 [13]. Following the termination of entomological and medical control operations in 1989, a total of 14 gHAT cases originating from the Vavoua tsetse control intervention area were diagnosed between 1991 and 1997 and no cases have been detected since 1997.

The situation in the Sinfra focus was different. Before tsetse control (October 1994), the mean daily catch of tsetse was 6.8 tsetse/trap and declined to only 3.7 tsetse/trap after 20 months of control (from November 1995 to July 1997). Prevalence of gHAT also decreased, from 0.69% in 1995 to 0.37% in 1997 [8, 9]. Despite tsetse still being relatively abundant, entomological and exhaustive active medical control operations terminated in 1997. Subsequently, a total of 228 gHAT cases were diagnosed originating from Sinfra tsetse control area between 1998 and 2015, and no cases have been detected since 2015.

In summary, in Vavoua, where the tsetse population was low (0.01 tsetse/trap/day), there was an interval of 8 years from the termination of tsetse and active medical control campaigns to the last gHAT case being detected into the tsetse control area, compared to 18 years for Sinfra where tsetse were less well controlled (3.7 tsetse/trap/day). This cessation of transmission in both locations indicate that a decline in transmission is likely to occur in the Bonon focus.

Following the large reduction in tsetse numbers, we planned to scale back the numbers of Tiny Targets deployed, especially in areas where tsetse were not caught during entomological monitoring and the T11 survey. Only 486 of the 2,003 Tiny Targets (24%) set up in February 2019 have been redeployed in February 2020, an additional 75 Tiny Targets were deployed in priority areas where the risk of contact between humans and tsetse is most likely. To limit reinvasion, 104 Tiny Targets have also been deployed strategically at the periphery of the intervention area, focussing on the river and track networks. Using this strategy, a total of 665 Tiny Targets have been deployed in February 2020, giving a density of ~7 targets/km^2^ or 1 target per 118 inhabitants.

This small-scale retrospective analysis of available data highlights that properly implemented *G*. *p*. *palpalis* control operation, in support to medical activities, can contribute to rapid interruption of *T*. *b*. *gambiense* transmission as demonstrated by the example of Vavoua [9]. In areas where tsetse are relatively abundant, sole reliance on active and passive screening interventions are unlikely to rapidly eliminate gHAT, as showed in Guinea and Chad [20, 21]. Our results suggest that use of Tiny Targets can help Côte d’Ivoire to achieve and maintain the goal of eliminating gHAT, by interrupting transmission of trypanosomes, including *T*. *b*. *gambiense*. Tiny Targets have now been incorporated as an important tool against tsetse flies in Côte d’Ivoire’s national strategy, acting in compliment to medical and veterinary control actions, combining to stop trypanosome transmission to humans and animals. Following gHAT case diagnosis Tiny Targets are now used in reactive deployed at locations near the patient’s residence where tsetse are likely to bite humans, these sites are identified during case follow-up activities.

## Supporting information

S1 Data Data file. (XLSX)
